# RETRACTION: Effectiveness of Nursing Intervention in the Operating Room to Prevent Pressure Ulcer and Wound Infection in Patients Undergoing Intertrochanteric Fracture: A Meta‐Analysis

**DOI:** 10.1111/iwj.70292

**Published:** 2025-03-03

## Abstract

**RETRACTION:** QiuF.‐F. and HuangS.‐M. , “Effectiveness of Nursing Intervention in the Operating Room to Prevent Pressure Ulcer and Wound Infection in Patients Undergoing Intertrochanteric Fracture: A Meta‐Analysis,” International Wound Journal 21, no. 2 (2024): e14434, 10.1111/iwj.14434.PMC1082873137849027

The above article, published online on 17 October 2023, in Wiley Online Library (http://onlinelibrary.wiley.com/), has been retracted by agreement between the journal Editor in Chief, Professor Keith Harding; and John Wiley & Sons Ltd. Following an investigation by the publisher, all parties have concluded that this article was accepted solely on the basis of a compromised peer review process. The editors have therefore decided to retract the article. The authors did not respond to our notice regarding the retraction.

**RETRACTION:** F.‐F. Qiu and S.‐M. Huang , “Effectiveness of Nursing Intervention in the Operating Room to Prevent Pressure Ulcer and Wound Infection in Patients Undergoing Intertrochanteric Fracture: A Meta‐Analysis,” International Wound Journal 21, no. 2 (2024): e14434, 10.1111/iwj.14434.PMC1082873137849027

The above article, published online on 17 October 2023, in Wiley Online Library (http://onlinelibrary.wiley.com/), has been retracted by agreement between the journal Editor in Chief, Professor Keith Harding; and John Wiley & Sons Ltd. Following an investigation by the publisher, all parties have concluded that this article was accepted solely on the basis of a compromised peer review process. The editors have therefore decided to retract the article. The authors did not respond to our notice regarding the retraction.

